# New Insights from the 7th World Melioidosis Congress 2013

**DOI:** 10.3201/eid2007.131737

**Published:** 2014-07

**Authors:** Herbert P. Schweizer, Direk Limmathurotsakul, Sharon J. Peacock

**Affiliations:** Colorado State University, Fort Collins, Colorado, USA (H.P. Schweizer);; Faculty of Tropical Medicine, Mahidol University, Bangkok, Thailand (D. Limmathurotsakul);; University of Cambridge, Cambridge, UK (S.J. Peacock)

**Keywords:** World Melioidosis Congress 2013, Burkholderia pseudomallei, melioidosis, current challenges, environmental saprophyte, bacterium, resource sharing

*Burkholderia pseudomallei* and the disease that it causes, melioidosis, have gained increasing attention over the last decade, largely because of the biothreat agenda. The increased attention has led to a boost in research funding and invigorated research efforts worldwide. The 7th World Melioidosis Congress 2013 (WMC2013), held in Bangkok, Thailand, on September 18–20, 2013, was attended by 321 delegates from 24 countries and featured 73 oral presentations and 157 poster presentations. The Congress documented impressive progress in regard to fundamental biological questions and the emerging distribution, epidemiology, clinical management, and prevention of melioidosis; selected key findings are shown in the Figure.

WMC2013 delegates highlighted current challenges for the melioidosis research community. The 2 most pressing clinical needs are to reduce the global incidence of melioidosis and improve the clinical outcome of infected persons. A 2-pronged approach will likely be needed to reduce incidence: vaccine development plus prevention of infection through behavior modification to reduce exposure-prone activities (e.g., farming without protective clothing and drinking untreated water). Challenges to the development of an effective vaccine include achieving sterilizing immunity, protecting from multiple routes of infection (i.e., ingestion, inhalation, and inoculation), and invoking an immune response to an opportunistic disease in persons with risk factors for infection (*15*). Such issues were addressed during a session devoted to vaccine development. It is clear that much research remains to be done before a viable melioidosis vaccine candidate emerges.

*B. pseudomallei* is an environmental saprophyte, and infection occurs through ingestion, inhalation, or inoculation of the organism (*16*); lowering the exposure risk should reduce disease incidence. Our understanding of the global distribution of environmental *B. pseudomallei* is incomplete, although several WMC2013 presentations confirmed that the pathogen is more widespread in tropical and subtropical regions of the world than previously thought; new endemic foci have been reported in multiple regions of Africa and Puerto Rico. The increasing detection of this pathogen in the environment emphasizes the ongoing need for a working group tasked with assessing inexpensive and simple methods for the detection of environmental *B. pseudomallei*. Such a group has already established consensus guidelines on environmental sampling of *B. pseudomallei* (*2*) and on an updated global distribution map (http://www.melioidosis.info/map.aspx). *B. pseudomallei* habitat studies in diverse environments in northern Australia are contributing to this work, as is a *B. pseudomallei* whole-genome sequencing project of several hundred isolates, both of which will provide an accurate picture of bacterial phylogeography.

The mapping of human and animal melioidosis cases is also essential to the development and implementation of control measures. The number of countries reporting endemic melioidosis continues to expand, and the number of melioidosis cases reported worldwide continues to increase. The reasons for these increases are probably multifactorial and include rising disease awareness among physicians, improved diagnostic capabilities, and increasing numbers of persons with risk factors. A major risk factor for melioidosis is diabetes (*16*). The numbers of persons with diabetes are increasing in many countries, causing concern that melioidosis may become even more prevalent. New models that predict the global distribution and incidence of melioidosis were described at WMC2013; these models will contribute to more accurate prevalence estimates. 

Improved outcome from melioidosis requires awareness, rapid diagnosis, and appropriate antimicrobial drug therapy. The lack of disease awareness among the public remains a major challenge, even in some countries with a high prevalence of disease. For example, Thailand is a major hotspot for melioidosis, yet public awareness of the disease is low. A survey of >4,200 persons in Thailand showed that only 26% had heard of melioidosis, and only 7% of those respondents knew what melioidosis is. There is an obvious need for risk communication, including education about daily activities that pose an exposure risk (*10*). 

Diagnosing melioidosis is challenging because clinical signs of the disease are nonspecific and varied, and a definitive diagnosis depends on the availability of diagnostic microbiology facilities. Advances are being made in developing and implementing novel diagnostic methods, such as antigen-based diagnostic tests for the detection of *B. pseudomallei* in body fluids and culture media. WMC2013 attendees also reiterated the importance of resource sharing of diagnostic reagents, including a latex agglutination test for the identification of *B. pseudomallei* isolated from culture media. This test is simple and suitable for resource-poor settings, yet is not widely available (*17*).

Continued efforts are also required to improve melioidosis treatment, including the streamlining of initial intravenous antimicrobial drug therapy and subsequent oral drug therapies and new drug evaluations. The publication of a study that confirms that doxycycline is not required for the oral phase of melioidosis treatment with trimethoprim-sulfamethoxazole (*3*) was an important step toward improving treatment. There was also discussion about the possibility of reducing the duration of oral therapy if preceded by more prolonged intravenous antimicrobial drug therapy. Current melioidosis therapeutic options are largely limited to β-lactam drugs (e.g., ceftazidime and meropenem) and trimethoprim-sulfamethoxazole (*4*). Antimicrobial drugs developed for other gram-negative bacteria are being evaluated for in vitro and in vivo efficacy in animal models, but none are currently being considered for clinical trials in persons with melioidosis. Other WMC2013 presentations described the basis for clinically significant antimicrobial drug–resistance mechanisms (*7*) and the evaluation of novel drug targets.

Our rapidly improving understanding of the biology and pathogenesis of *B. pseudomallei* underpins the clinical aspirations for prevention, diagnosis, and improved patient outcome. As in other disciplines, whole-genome sequencing is being used in this field to ascertain pathogen evolution and adaptation to the host environment in chronically infected patients, such as persons with cystic fibrosis and non–cystic fibrosis bronchiectasis (*18*). The collective findings of numerous studies are unraveling host–pathogen interactions and the role of innate immunity during initial infection. Together with findings from studies of comparative *B. pseudomallei* gene expression in humans and murine hosts, such findings are becoming more clinically relevant. The report of various diabetic mouse models and their use for studying the pathogenesis of melioidosis reflects the significance of diabetes as a risk factor and our lack of knowledge in this area.

Notable progress was described at WMC2013 in basic and applied research concerning melioidosis and its bacterial cause, yet many challenges remain. Continued progress will undoubtedly be presented at the next WMC, scheduled for 2016 in Honolulu, Hawaii, USA.

**Figure:**
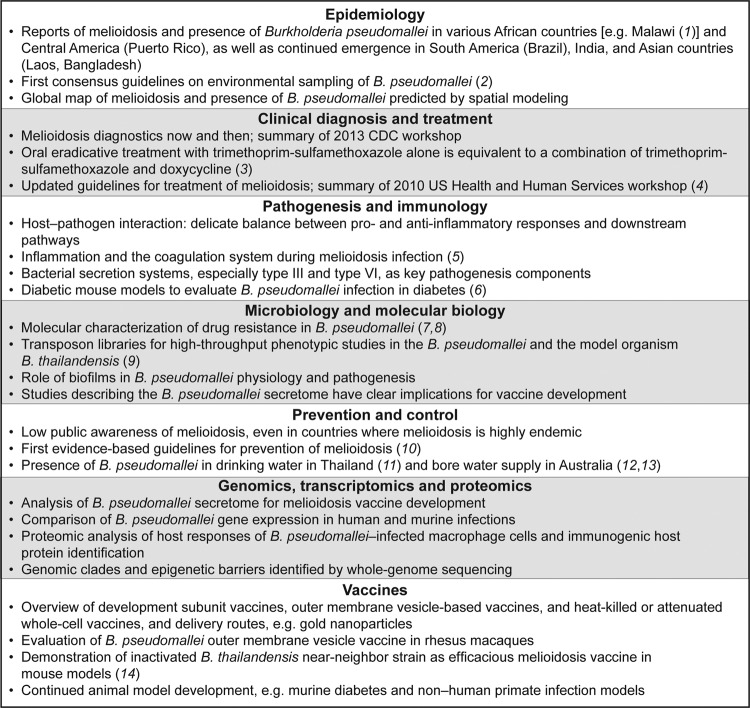
Selected key findings presented at the 7th World Melioidosis Congress 2013 in Bangkok, Thailand, September 18–20, 2013.
